# FedCMC: a federated learning model with contribution fairness based on multi-center core data extraction for assessing the myometrial invasion status of endometrial cancer

**DOI:** 10.3389/fonc.2025.1648502

**Published:** 2025-09-09

**Authors:** Yuping Li, Bao Feng, Yuan Chen, Xiaohong Ruan, Jiangfeng Shi, Ximiao Wang, Xianyan Wen, Peijun Li, Junqi Sun, Changye Zheng, Yujian Zou, Mingwei Li, Wansheng Long, Yehang Chen, Dong Xie

**Affiliations:** 1 School of Electronic Engineering and Automation, Guilin University of Electronic Technology, Guilin, Guangxi, China; 2 Laboratory of Intelligent Detection and Information Processing, Guilin University of Aerospace Technology, Guilin, Guangxi, China; 3 Jiangmen Key Laboratory of Artificial Intelligence in Medical Image Computation and Application, Jiangmen Central Hospital, Jiangmen, Guangdong, China; 4 Department of Gynecology, Jiangmen Central Hospital, Jiangmen, Guangdong, China; 5 Clinical Transformation and Application Key Lab for Obstetrics and Gynecology, Pediatrics, and Reproductive Medicine of Jiangmen, Jiangmen Central Hospital, Jiangmen, Guangdong, China; 6 School of Automation Science and Engineering, South China University of Technology, Guangzhou, Guangdong, China; 7 Department of Radiology, Jiangmen Central Hospital, Jiangmen, Guangdong, China; 8 Department of Radiology, Yuebei People’s Hospital, Shaoguan, Guangdong, China; 9 Department of Radiology, Affiliated Dongguan Hospital, Southern Medical University, Dongguan, Guangdong, China; 10 Department of Gynecology, Kaiping Central Hospital, Kaiping, Guangdong, China; 11 College of Science, Guilin University of Aerospace Technology, Guilin, Guangxi, China

**Keywords:** federated learning, fairness, core data extraction, endometrial cancer, myometrial invasion, personalized treatment strategies

## Abstract

**Background:**

Multi-center Federated Learning (FL) has played a significant role in disease prediction, offering a feasible solution to the challenges of cross-institutional collaboration. However, the fairness issues inherent in traditional FL frameworks have limited their further development in the medical field.

**Methods:**

We propose a Contribution Fairness Federated Learning model based on Multi-center Core Data Extraction (FedCMC). This model accurately assesses the actual contributions of each center from both data and model perspectives using two fairness indicators: data information richness and model quality. In the data contribution assessment phase, we innovatively design a Multi-center Core Data Extraction Module (MCDEM). This module extracts representative core datasets from the original training pool, effectively filtering redundant information and enhancing the fairness of data contribution assessment and the model's generalization ability. Subsequently, weighted aggregation based on each center's contribution optimizes the benefits for high-contribution centers, incentivizing more users to participate in federated learning. Finally, a personalized federated learning strategy is adopted, enabling the model to fine-tune through each center's core dataset, thereby improving its prediction relevance and accuracy.

**Results:**

We analyze data from 902 endometrial cancer (EC) patients across four independent medical institutions. In centers A, B, and C, the FedCMC model achieves areas under the ROC curve (AUC) of 0.8261, 0.8750, and 0.8964, respectively. Comparative analysis with three traditional federated learning algorithms indicates that FedCMC offers significant advantages in both performance and fairness.

**Conclusion:**

FedCMC effectively alleviates fairness issues in traditional FL frameworks and accurately predicts the myometrial invasion (MI) status of EC patients, supporting personalized treatment strategies.

## 1 Introduction

Endometrial cancer (EC) was the sixth most commonly diagnosed cancer among women worldwide in 2022, with its incidence and mortality rates continuing to rise. Reported deaths increased from 97,370 in 2020 to 97,704 in 2022, posing a significant threat to women’s health (1, 2). EC is typically treated through hysterectomy (3). While this approach has improved patient survival rates, conservative methods are also safe and feasible for some patients with early-stage EC (4). Assessing the status of myometrial invasion (MI), which determines whether the tumor is confined to the endometrium or has invaded the myometrium, is crucial for risk stratification and helps develop personalized treatment plans (5). According to the 2021 ESGO guidelines, the choice of surgical staging in specific cases depends on MI status (6). Research further suggests that preoperative determination of MI status aids in selecting optimal treatment strategies, especially for younger EC patients wishing to preserve fertility (7). Thus, gynecologists urgently need to confirm MI status before treatment to adjust therapeutic approaches, improve patient outcomes, and optimize healthcare resources.

In recent years, deep learning technologies have been widely applied in the medical field, achieving notable results (8–10). As a data-driven technology, deep learning models trained on single-center medical data are often limited in scale, and their applicability is confined to specific scenarios, resulting in relatively poor generalization capabilities (11). To develop stable and effective general artificial intelligence models, multi-center research has become crucial. However, due to regulations on data privacy protection, data from different medical institutions in multi-center studies cannot be directly shared (12). Federated learning, as a distributed machine learning framework, enables the extraction of feature representations from different centers without sharing private data, offering a practical solution for cross-center collaboration in the medical field (13, 14).

Nevertheless, with the continuous growth in data scale, the accumulation of redundant information poses challenges to both model training efficiency and performance enhancement. The key issue now is how to extract a representative core dataset from the massive amount of data to improve the training or fine-tuning of models. For example, in multi-center federated learning scenarios, how to rapidly extract a core dataset for training and personalized fine-tuning of models; or in complex application contexts, how to leverage a small core dataset to help large models quickly adapt to task requirements, are pressing issues that need to be addressed.

Ensuring sufficient client participation is key to guaranteeing the performance of federated learning models (15, 16). In a federated learning framework involving multi-party collaboration, the contributions of different participants to the learning process may vary significantly. These contributions are influenced by factors such as data scale and data quality. However, current federated learning systems often lack fair contribution evaluation. Some clients possess large-scale data and abundant local computational resources, resulting in high-quality uploaded models. Nevertheless, these contributions are not appropriately valued by the server. When aggregated with lower-quality local models, the overall model performance may degrade, and the distributed model may even perform worse than the local models (17). Moreover, some federated frameworks overly prioritize data scale while neglecting data quality, leading to clients with high-quality but small-scale data being overlooked (18). This neglect of contributors’ efforts undermines their motivation to participate actively in federated learning. Therefore, to attract more participants to federated learning, it has become imperative to develop a framework that can fairly reflect the contributions of each participant.

To address the aforementioned challenges, this study proposes a Contribution-Fair Federated Learning Model Based on Multi-Center Core Data Extraction (FedCMC). First, we comprehensively evaluate the contribution of each center from both data and model perspectives. Specifically, we design an innovative Multi-Center Data Extraction Mechanism (MCDEM) to extract the core dataset, where data contribution is assessed based on the richness of local data information rather than simply relying on data volume. Second, based on the contribution evaluation results, we perform weighted aggregation to optimize the benefits of high-contribution centers, thereby incentivizing active participation in federated learning and enhancing collaborative motivation. Finally, to mitigate model performance inconsistencies caused by data heterogeneity across centers, we adopt a personalized federated learning strategy (19). By fine-tuning the global model on the extracted core dataset, we further improve model performance across different centers and alleviate performance disparities. Through this approach, we achieve a fairer and more accurate prediction of myometrial invasion status in EC patients, providing technical support for clinical strategy optimization and facilitating more targeted and personalized treatment plans.

## 2 Materials and methods

### 2.1 Patients

This retrospective study was approved by the ethics committees of the four participating centers. Due to its retrospective nature, informed consent was waived. The study reviewed the medical records of 757 patients diagnosed with endometrial cancer (EC) via hysterectomy and confirmed by pathology at Center A between September 2010 and September 2022, along with their corresponding MRI imaging data. Additionally, Centers B, C, and D reviewed relevant data for 459, 374, and 40 patients, respectively, between December 2016 and February 2023. Given the small sample size at Center D, the data from Center D were merged into Center C for subsequent analyses.

**Figure 1:**
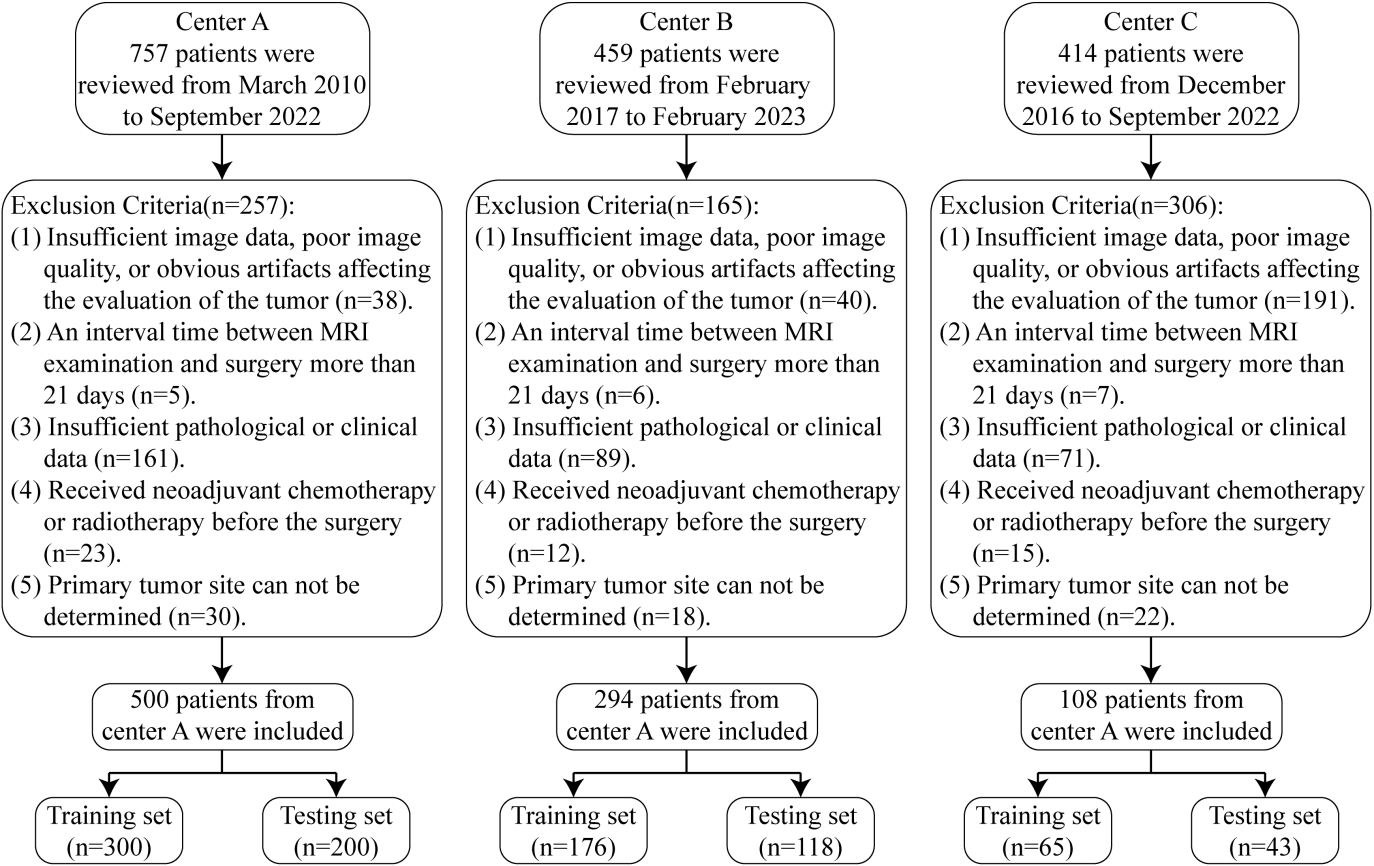
Patient inclusion and exclusion process.

The inclusion criteria were as follows: (1) uterine malignant epithelial tumors confirmed by total hysterectomy (including EEC, serous carcinoma, clear cell carcinoma, undifferentiated and dedifferentiated carcinoma, mixed carcinoma, and carcinosarcoma); (2) MRI examination containing sagittal T2-weighted imaging (T2WI) sequences; (3) pelvic MRI completed within 21 days before surgery; (4) complete clinical and pathological information. Exclusion criteria were: (1) poor image quality or significant artifacts affecting tumor assessment; (2) MRI performed more than 21 days before surgery; (3) insufficient pathological or clinical data; (4) patients who received neoadjuvant chemotherapy or radiotherapy prior to surgery; (5) inability to determine the location of the primary tumor. The patient inclusion and exclusion flow for the three centers is shown in **Figure 1**.

The clinical characteristics of each included patient were categorized into two groups. The first group comprised clinicopathological features, including age and post-hysterectomy pathological results such as histopathological type, grade, lymphovascular space invasion (LVSI) status, myometrial invasion (MI) status, and FIGO stage (2009) (20). The second group included subjective MRI assessments, such as the maximum tumor diameter (TMD) observed on MRI and the radiologists’ evaluation of MI status. Ultimately, 812 EC patients were included in this multi-center study. Patients from Centers A, B, and C were 410, 294, and 108, respectively, and were randomly divided into training and test sets in a 6:4 ratio. Basic patient information is detailed in **Table 1**.

**Table 1:** Basic patient information.

Center	Dataset	Status	Age (mean ± std)	TMD (mean ± std)	Histopathologic type		Histopathologic Grade			Read for MI status		FIGO stage			LVSI	
					EEC	Others	1	2	3	Absence	Presence	IA	IB	Others	Absence	Presence
Center A (500)	Train (300)	Non-MI(38)	47.76 ± 8.04	3.61 ± 1.89	35	3	25	9	4	9	29	36	0	2	37	1
		MI(262)	55.59 ± 8.06	4.33 ± 2.31	246	16	61	137	64	16	246	138	51	73	183	79
	Test (200)	Non-MI(25)	50.80 ± 8.63	2.76 ± 1.31	22	3	14	8	3	15	10	24	0	1	25	0
		MI(175)	55.13 ± 7.81	4.34 ± 2.17	153	22	33	92	50	15	160	87	37	51	113	62
Center B (294)	Train (176)	Non-MI(11)	51.27 ± 8.55	2.95 ± 2.59	11	0	11	0	0	3	8	11	0	0	11	0
		MI(165)	54.43 ± 8.07	4.05 ± 2.16	151	14	74	62	29	15	150	102	29	34	126	39
	Test (118)	Non-MI(8)	50.88 ± 10.19	3.70 ± 2.15	7	1	7	0	1	1	7	7	0	1	8	0
		MI(110)	54.84 ± 7.93	3.54 ± 1.59	98	12	49	38	23	13	97	70	17	23	93	17
Center C (108)	Train (65)	Non-MI(10)	46.20 ± 9.14	2.86 ± 1.60	10	0	10	0	0	2	8	10	0	0	10	0
		MI(55)	54.07 ± 9.04	4.27 ± 1.97	52	3	27	18	10	4	51	31	12	12	45	10
	Test (43)	Non-MI(6)	53.50 ± 9.85	4.05 ± 1.74	5	1	3	2	1	3	3	6	0	0	5	1
		MI(37)	60.11 ± 9.11	4.06 ± 1.97	35	2	8	23	6	9	28	20	5	12	36	1

Non-MI refers to endometrial cancer without myometrial invasion, MI refers to endometrial cancer with myometrial invasion; TMD, maximum diameter of the tumor on MRI images; mean indicates the average value, and std represents the standard deviation; Histopathologic type-EEC, endometrial endometrioid carcinoma; Histopathologic type-others, serous carcinoma, clear cell carcinoma, undifferentiated and dedifferentiated carcinoma, mixed carcinoma and carcinosarcoma; Reading for MI, radiologist’s assessment of myometrial invasion; LVSI-Absence, negative lymphovascular space invasion; LVSI-Presence, positive lymphovascular space invasion.

### 2.2 MRI protocol and definition of MI

MRI examinations were performed using 1.5 or 3.0 T scanners, with patients positioned supine and breathing freely (see **Supplementary Tables S1**-**S4** for MRI parameters). Two radiologists with specialized training in gynecologic imaging (with over 8 and 10 years of experience, respectively) independently reviewed the images, assessing the maximum tumor diameter and MI status on MRI. The radiologists were blinded to the patients’ postoperative pathological results, and disagreements were resolved through discussion.

On T2WI images, EC was defined as a localized endometrial lesion with signal intensity (SI) lower than normal endometrium but higher than the myometrium (21). MI status was categorized as no MI or MI, with MI further divided into superficial invasion (involving <50% of the myometrium) and deep invasion (involving ≥50% of the myometrium). The integrity of the junctional zone (JZ) was a critical criterion for MI assessment: disruption or interruption of the JZ indicated MI, whereas an intact JZ suggested no MI (22).

The surgical pathological diagnosis was performed by experienced clinical specialists in gynecological pathology. Pathological type, differentiation grade, MI status, and LVSI status were determined based on the 2020 WHO Classification of Tumors of Female Reproductive Organs. Staging was conducted according to the 2009 FIGO guidelines. Any diagnostic discrepancies were resolved through discussion.

### 2.3 ROI delineation

Regions of interest (ROI) were defined by a radiologist with >10 years of experience in gynecological imaging diagnosis. The delineation aimed to accurately contour the lesion. Subsequently, a rectangular bounding box covering the lesion’s boundary was constructed, minimizing the impact of subjective ROI selection by clinicians (23, 24). Deep learning methods were utilized to automatically extract ROI, eliminating the need for precise manual delineation. Details of the data preprocessing steps are provided in **Supplementary Material S1**.

### 2.4 Construction of the FedCMC framework

**Figure 2:**
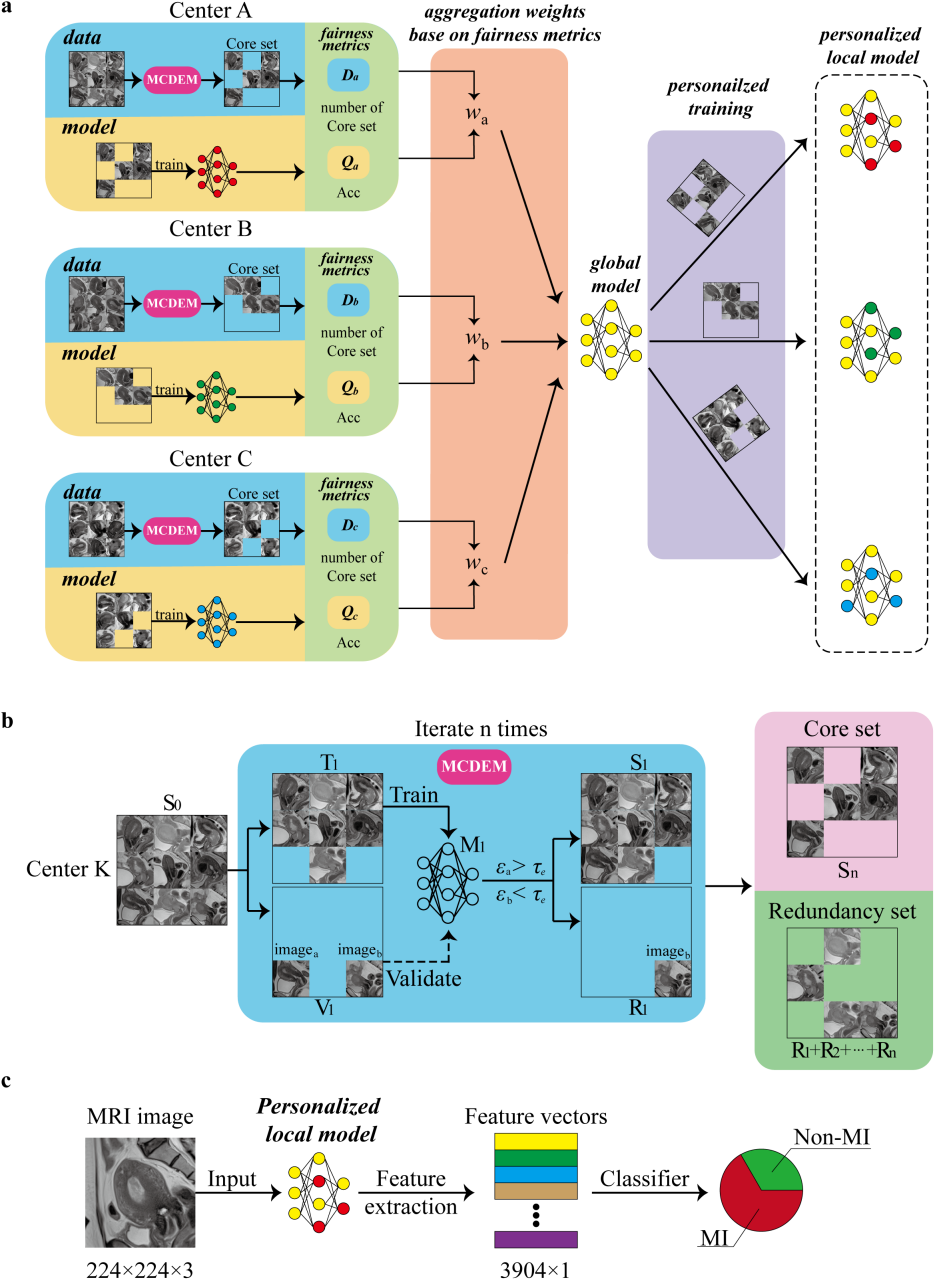
Framework of the FedCMC algorithm. **(a)** The process of constructing personalized local models in FedCMC. **(b)** Screening mechanism of the multi-center core data extraction module (MCDEM). **(c)** Feature extraction and classification in FedCMC. *D* represents the data richness evaluation metric; *Q* denotes the model quality evaluation metric; *w* is the contribution fairness aggregation weight; and S refers to the original training dataset; T and V represent the training and validation datasets obtained by randomly splitting S in an 8:2 ratio;is the prediction error of the samples in the validation set;is the error threshold used to identify redundant data (R); Non-MI endometrial cancer without myometrial invasion; MI endometrial cancer with myometrial invasion. A model is trained on the dataset T, and its prediction error is evaluated on the validation dataset V.

To address the fairness challenges faced by medical centers in federated learning, this study proposes a Contribution Fairness Federated Learning model based on Multi-center Core Data Extraction (FedCMC). First, we conduct a comprehensive evaluation of each center’s contributions from both data and model perspectives. Given that varying degrees of information redundancy exist in local data across centers, directly using such data may lead to model bias and unfair data contribution assessment. To address this, we designed a Data Pruning Module to identify a core set of samples that represent the richness of each center’s data information. These core samples are used to train local models and fairly evaluate data contributions. Additionally, the model’s accuracy, which intuitively reflects its quality, is incorporated to evaluate the overall contribution. Second, to address differences in contributions among centers, we introduce contribution-based fairness aggregation to optimize the actual benefits for high-contribution centers, thereby enhancing collaboration fairness across institutions. Finally, we adopt a personalized federated learning strategy to mitigate inconsistencies in global model performance across different centers. The algorithmic framework of FedCMC is illustrated in **Figure 2**.

#### 2.4.1 Contribution evaluation based on model quality and core data

To accurately evaluate the contributions of each center, we propose two fairness metrics: data information richness and model quality.

Data information richness is measured by the size of the core sample set at each center. Since local data at different centers often contain varying degrees of redundancy, directly using such data can lead to model bias and unfair evaluation of data contributions. We designed a Data Pruning Module to eliminate samples rich in redundant information, retaining only core samples that represent the richness of each center’s data. This approach shifts the data distribution toward underrepresented information, enhancing the model’s adaptability to different categories of data and improving its generalization ability. Details of the Data Pruning Module can be found in **Supplementary Material S2**.

Model quality is primarily assessed based on the accuracy of each center’s local model. Specifically, the accuracy of local models during each training round serves as the evaluation metric. This measure directly reflects how well a center’s model adapts to and performs on its own data. By focusing on higher-quality local models during global aggregation, the global model’s performance can be optimized while minimizing the adverse effects of low-quality models.

#### 2.4.2 Contribution-based fair aggregation

We optimize the rewards for high-contribution centers through fair aggregation to motivate centers to actively participate in federated learning. Due to differences in data scale and quality among centers, their contributions to the global model vary. If high-contribution centers and low-contribution centers receive the same level of performance rewards, it would be unfair to the high-contribution centers, potentially discouraging their participation.

To address this issue, we propose an adaptive weighted aggregation method based on the contribution of each center. Contribution-weighted aggregation allows the global model to better fit the data distribution of high-contribution centers, improving its performance at these centers and increasing their rewards. At the same time, high contributions typically indicate higher-quality local models and richer data sources. Assigning greater weights to high-contribution centers not only enables the global model to learn more task-relevant features but also reduces the negative impact of low-performance centers, ultimately leading to a stronger global model. Details of the aggregation process are provided in **Supplementary Material S3**.

#### 2.4.3 Personalization strategy

To address the issue of inconsistent global model performance caused by data heterogeneity among centers, we adopt a personalized federated learning strategy. Specifically, the global model is fine-tuned on each center’s local data, resulting in a personalized model that better aligns with the local data distribution. This strategy effectively enhances the performance of models at all centers, particularly for low-contribution centers and those with highly heterogeneous data.

To maximize feature utilization, each center used its local model’s convolutional kernels as feature extractors, extracting multiple feature maps from MRI images for each patient. The mean of these feature maps was calculated to create unified deep learning features (**Supplementary Figure S3**). A total of 3904 deep learning features were extracted using the 3904 convolutional kernels, which were then used to construct a classifier (**Supplementary Figure S4**). SBELM (25) integrates an L1-norm into the optimization of the extreme learning machine to automatically select the most informative features, yielding a sparse solution. In high-dimensional, small-sample medical settings, it demonstrates superior generalization performance compared with other classifiers; therefore, SBELM was chosen as the classifier for this study (classifier comparison in **Supplementary Table S5**). To address the class imbalance in this binary classification problem, focal loss (26) was used for all loss functions to alleviate potential bias caused by the imbalance between positive and negative samples.

### 2.5 Model evaluation and comparison

To comprehensively evaluate the performance of FedCMC in a multi-center setting, this study conducted comparative analyses with three classic federated learning models: FedAvg (18), FedProx (27), and Moon (28). To systematically assess the performance of each algorithm, quantitative metrics such as the AUC, specificity, sensitivity, accuracy (ACC), area under the Precision-Recall curve (PR-AUC), and F1-score were employed to validate the predictive outcomes of the models. The AUC metric was calculated with a 95% confidence interval (CI) to more accurately measure the robustness of the models across different datasets. These evaluation metrics provide a comprehensive perspective on the predictive capabilities of the algorithms and offer an objective basis for statistical comparisons between models. Additionally, decision curve analysis (DCA) was used to assess the clinical utility of the models in predicting MI status in endometrial cancer.

### 2.6 Statistical analysis

Statistical analyses were conducted using two-tailed tests, with a p-value < 0.05 considered statistically significant. All analyses were performed using R software (version 4.2.2) and IBM SPSS Statistics (version 26.0).

## 3 Result

### 3.1 FedCMC prediction of myometrial invasion in endometrial cancer patients

The experimental results demonstrate that the proposed FedCMC algorithm achieved high diagnostic performance across centers A, B, and C (**Table 2**). The threshold corresponding to the maximum Youden index was chosen as the optimal diagnostic threshold for the model of each center. Subsequently, we used these thresholds to calculate other performance metrics for each center. The sizes of the pruned training datasets in centers A, B, and C were 57.4%, 43.5%, and 88.7% of their original sizes, respectively. Using only the pruned training data, the models achieved AUC values of 0.8238, 0.8830, and 0.9130 on the test sets for centers A, B, and C, respectively.

**Table 2:** Diagnostic performance of FedCMC in multiple centers.

Center	DataSet	Radio	AUC (95% CI)	Accuracy	Sensitivity	Specificity	PR-AUC	F1
A	Training Cohort (n=164)	57.4% (2179/3795)	0.8429 (0.7674 - 0.9185)	0.8171 (134/164)	0.8254 (104/126)	0.7895 (30/38)	0.9312	0.8739
	test Cohort (n=200)	–	0.8261 (0.7343 - 0.9178)	0.7800 (156/200)	0.8000 (140/175)	0.6400 (16/25)	0.9583	0.8642
B	Training Cohort (n=74)	43.5% (843/1940)	0.9062 (0.8138 - 0.9986)	0.8919 (66/74)	0.9048 (57/63)	0.8182 (9/11)	0.9655	0.9344
	test Cohort (n=118)	–	0.8750 (0.7576 - 0.9924)	0.8729 (103/118)	0.8818 (97/110)	0.7500 (6/8)	0.9802	0.9282
C	Training Cohort (n=58)	88.7% (613/691)	0.9271 (0.8457 - 1.0000)	0.9138 (53/58)	0.9375 (45/48)	0.8000 (8/10)	0.9626	0.9474
	test Cohort (n=43)	–	0.8964 (0.7110 - 1.0000)	0.9535 (41/43)	0.9730 (36/37)	0.8333 (5/6)	0.9499	0.9730

Radio refers to the proportion of core dataset relative to the original training pool. For instance, in Center A, the pruned dataset contains 2,179 images compared to 3,795 images in the original dataset, accounting for 57.4%.

### 3.2 Comparison of FedCMC with other algorithms

**Figure 3:**
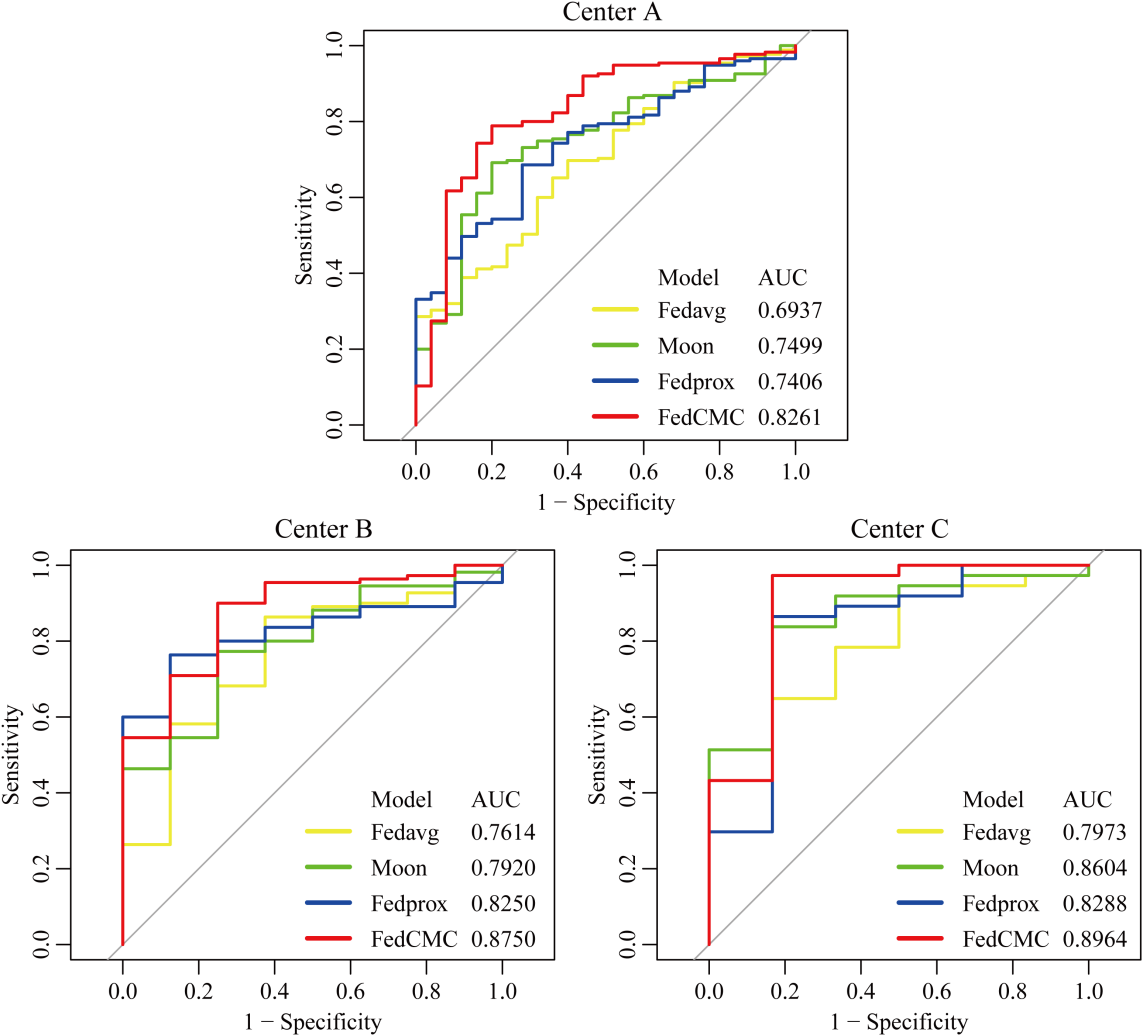
ROC curves of four algorithms across three centers. ROC receiver operating characteristic curve.

**Figure 4:**
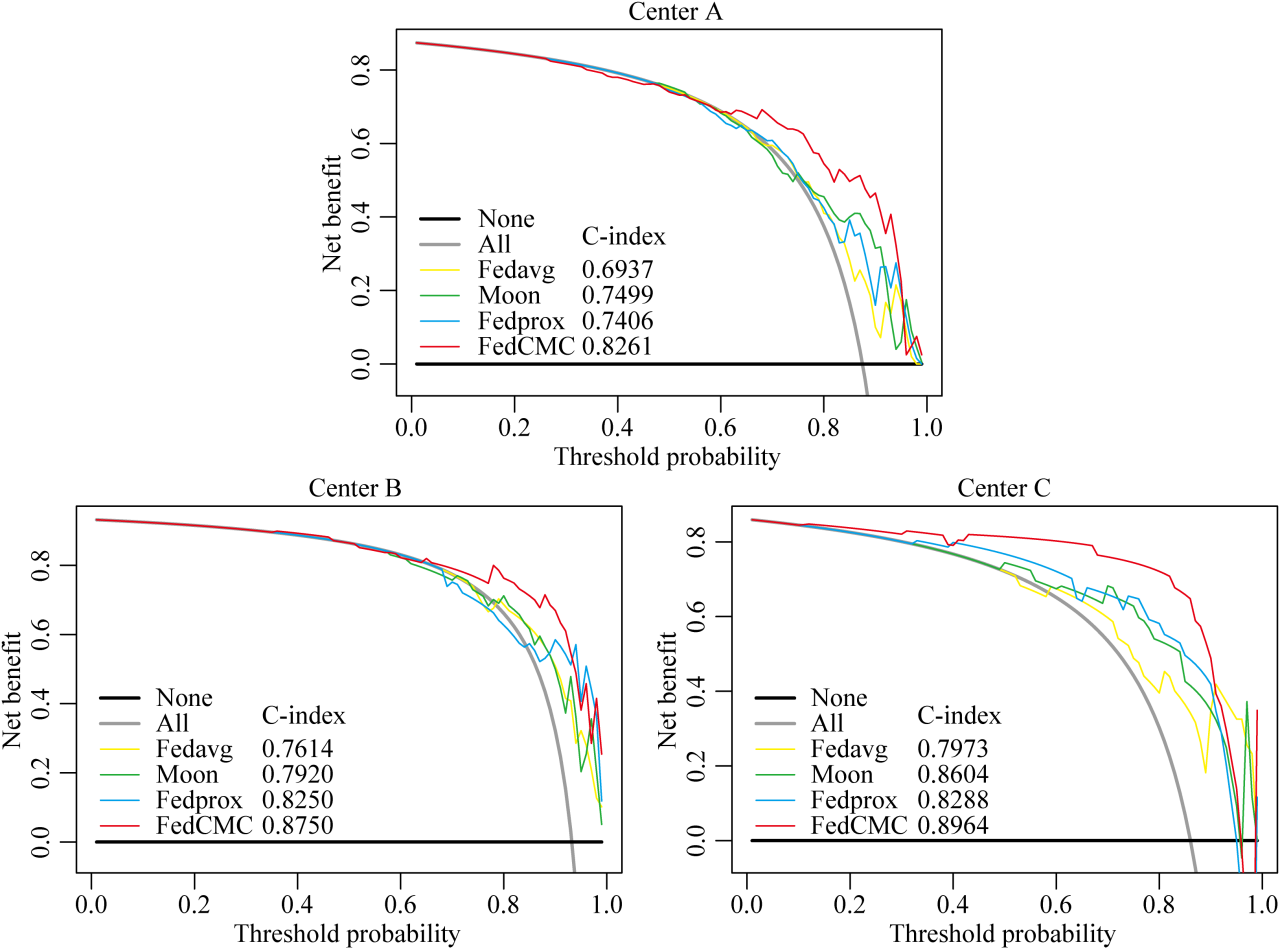
DCA curves of three models using data from three centers. The gray solid line represents the assumption that all patients belong to the MI group of endometrial cancer, while the black line assumes no patients belong to the MI group. Threshold probability represents the point where the expected benefits of treatment and avoidance of treatment are equal. FedCMC, ours algorithm; Fedavg, Moon, and Fedprox, comparison algorithms.

To further evaluate the performance of FedCMC, it was compared against three federated learning algorithms (Fedavg, Fedprox, and Moon). **Figures 3**, **4** illustrate the ROC curves (results of DeLong test, NRI, and IDI are provided in **Supplementary Table S6**) and DCA curves for the four algorithms across the three centers. FedCMC consistently achieved the highest AUC results in all three centers (**Table 3**). The results indicate that FedCMC outperformed the other three algorithms while using only 56.6% (3635/6426) of the training data, demonstrating the effectiveness of the proposed approach.

**Table 3:** Performance comparison of four algorithms on test sets of three centers.

Center	Algorithm	AUC (95% CI)	Accuracy	Sensitivity	Specificity	PR-AUC	F1
A	Fedavg	0.6937 (0.5900 - 0.7974)	0.7000 (140/200)	0.7314 (128/175)	0.4800 (12/25)	0.9372	0.8101
	Moon	0.7499 (0.6553 - 0.8446)	0.7150 (143/200)	0.7143 (125/175)	**0.7200** **(18/25)**	0.9477	0.8143
	Fedprox	0.7406 (0.6512 - 0.8300)	0.7500 (150/200)	0.7714 (135/175)	0.6000 (15/25)	0.9492	0.8438
	**FedCMC**	**0.8261** **(0.7343 - 0.9178)**	**0.7800** **(156/200)**	**0.8000** **(140/175)**	0.6400 (16/25)	**0.9583**	**0.8642**
B	Fedavg	0.7614 (0.5871 - 0.9356)	0.6525 (77/118)	0.6455 (71/110)	**0.7500** **(6/8)**	0.9671	0.7760
	Moon	0.7920 (0.6498 - 0.9343)	0.7091 (78/110)	0.7091 (78/110)	**0.7500** **(6/8)**	0.9725	0.8211
	Fedprox	0.8250 (0.7310 - 0.9190)	0.7881 (93/118)	0.7909 (87/110)	**0.7500** **(6/8)**	0.9770	0.8744
	**FedCMC**	**0.8750** **(0.7576 - 0.9924)**	**0.8729** **(103/118)**	**0.8818** **(97/110)**	**0.7500** **(6/8)**	**0.9802**	**0.9282**
C	Fedavg	0.7973 (0.6263 - 0.9683)	0.7209 (31/43)	0.7297 (27/37)	0.6667 (4/6)	0.9357	0.8182
	Moon	0.8604 (0.7028 - 1.0000)	0.8372 (36/43)	0.8649 (32/37)	0.6667 (4/6)	0.9468	0.9014
	Fedprox	0.8288 (0.6078 - 1.0000)	0.7907 (34/43)	0.7838 (29/37)	**0.8333** **(5/6)**	0.9336	0.8657
	**FedCMC**	**0.8964** **(0.7110 - 1.0000)**	**0.9535** **(41/43)**	**0.9730** **(36/37)**	**0.8333** **(5/6)**	**0.9499**	**0.9730**

Bold font indicates the best metric in each column for each center.

### 3.3 Ablation study of FedCMC

To validate the effectiveness of different components within FedCMC, ablation studies were conducted on the MCDEM and the fair aggregation mechanism. The specific AUC results are shown in **Table 4**.

**Table 4:** AUC results of the FedCMC ablation study.

MCDEM	Fair Aggregation	Data Set	Center A	Center B	Center C
		Train	0.7831	0.8562	0.9145
		Test	0.7355	0.8148	0.8649
✓		Train	0.8076	0.8470	0.9229
		Test	0.7721	0.8409	0.8874
✓	✓	Train	0.8429	0.9062	0.9271
		Test	0.8261	0.8750	0.8964

MCDEM, multi-center core data extraction module; Fair Aggregation, fairness-based weighted aggregation mechanism.

In the ablation experiments, the comparison between Group 1 and Group 2 demonstrates that the data pruning module effectively improves the model’s diagnostic performance by removing redundant data. The comparison between Group 2 and Group 3 indicates that the fairness aggregation mechanism further optimizes the global weight distribution of the model, leading to overall performance improvement across the four centers. Notably, the performance enhancement is more pronounced in high-contribution centers, aligning with the fairness principle that higher-contribution centers receive greater rewards. Meanwhile, the performance of low-contribution centers is not compromised. In the ablation experiments, when the fairness aggregation mechanism is not employed, the aggregation strategy defaults to average aggregation.

### 3.4 Analysis of FedCMC algorithm results

**Figure 5:**
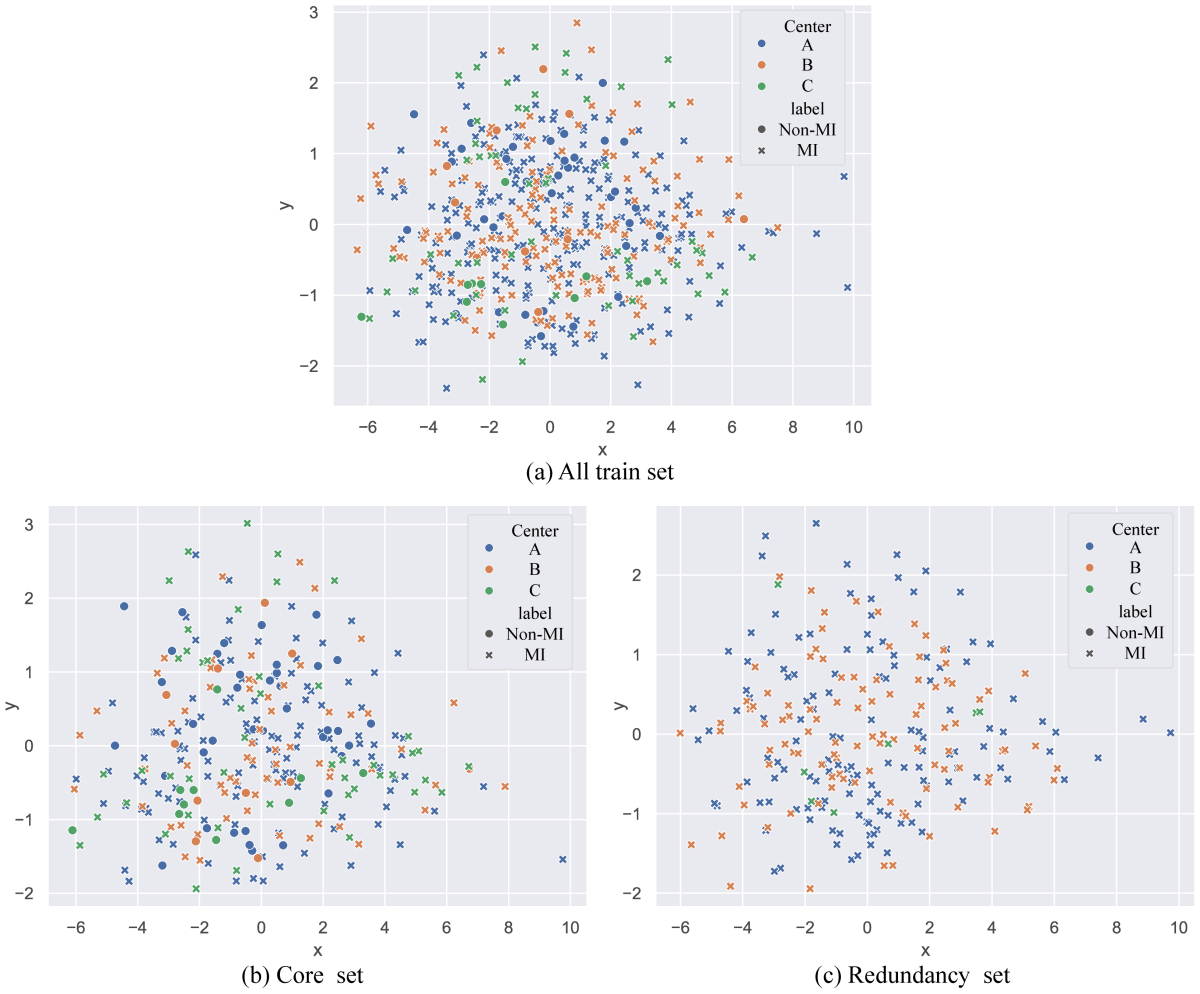
Scatter plots of training samples from three centers after PCA dimensionality reduction. **(a)** Spatial distribution of training samples in the original randomly split training sets of three centers. **(b)** The sample space distribution of the core dataset, representing the sample diversity of each center after pruning by the MCDEM. **(c)** The sample space distribution of the redundant dataset for each center after pruning by the MCDEM. Non-MI refers to endometrial cancer without myometrial invasion, and MI refers to endometrial cancer with myometrial invasion.

**Figure 6:**
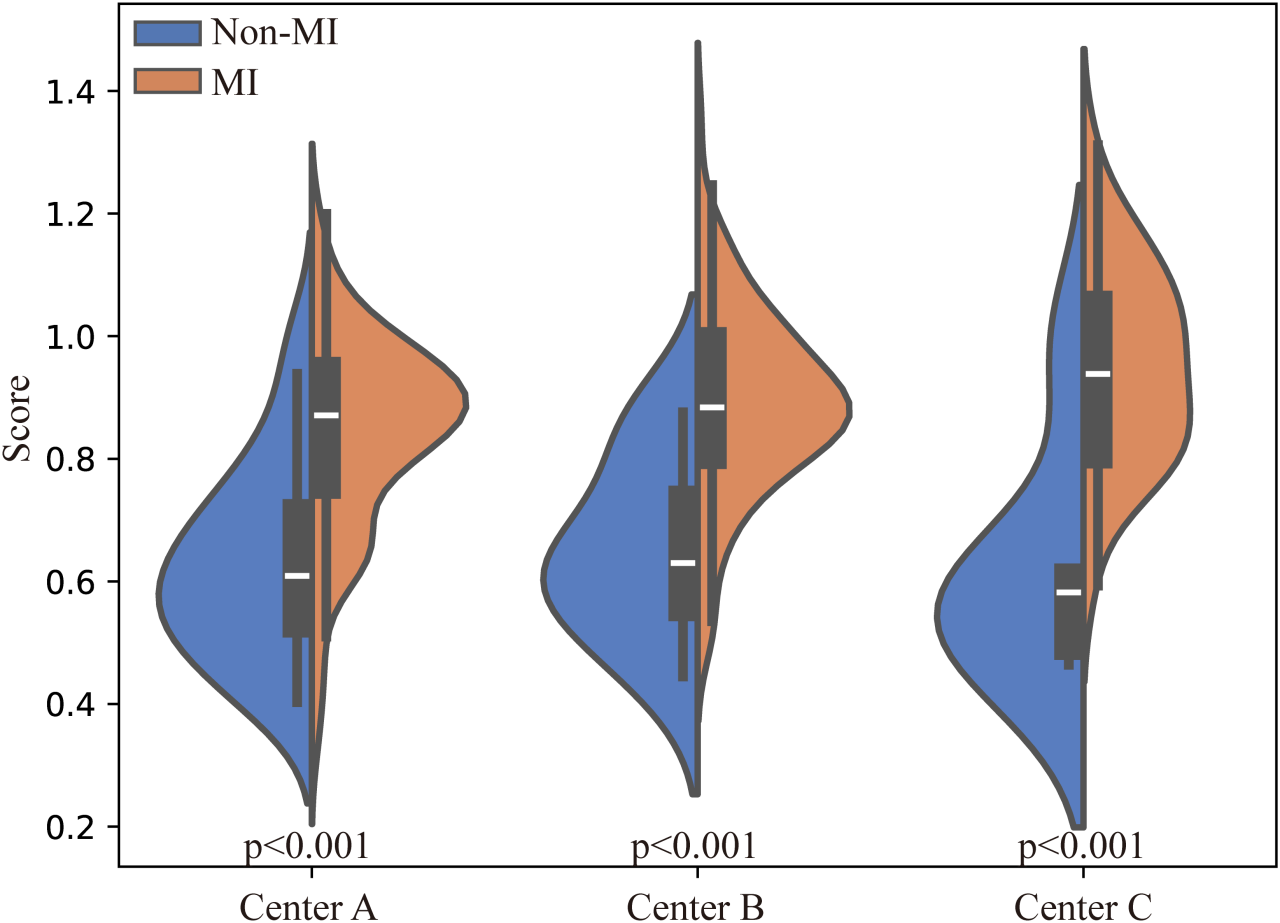
Violin plots. Illustrating the distribution of positive and negative samples in three data centers evaluated by FedCMC. Statistical test: independent t-test (two-tailed).Non-MI, no myometrial invasion; MI, myometrial invasion; p, significance value.

To visually observe the distribution of redundant samples in local data, we used Principal Component Analysis (PCA) to map the sample distributions into a two-dimensional space (29) (**Figure 5**). The overlapping distribution of redundant and original data indicates high similarity between them. This suggests that redundant samples may not provide additional information to the model, and their removal can mitigate model bias by reducing over-representation of repetitive information without compromising representational capacity. This visual comparison validates the effectiveness of our MCDEM, which preserves the core characteristics of the dataset while reducing redundancy, revealing the redundancy in local data at each center. **Figure 6** illustrates the prediction scores of FedCMC for two categories, with p < 0.05 indicating significant differences in the model’s predictions between the two labels. This demonstrates the model’s effectiveness in distinguishing between MI and Non-MI statuses of EC patients.

## 4 Discussion

In recent studies on endometrial cancer prediction, Coada et al. extracted 107 radiomics features from contrast-enhanced CT scans of 81 patients in a single-center cohort and employed LASSO-Cox, CoxBoost, and random forest survival models to stratify postoperative recurrence risk, achieving an AUC of 0.86 – 0.90 on the test set (30). Li et al. utilized a multi-center cohort of 415 patients, integrating T2-weighted MRI radiomics features with clinical information to construct a multi-classification model for preoperative prediction of deep myometrial invasion, high-risk classification, histological subtype, and lymphovascular space invasion (LVSI), with test-set AUCs ranging from 0.79 to 0.91 (31). However, single-center studies often suffer from limited sample sizes, making it challenging to train models that are both robust and generalizable. Moreover, centralized training is difficult to implement in practice due to medical data privacy concerns, highlighting the importance of conducting multi-center collaborative studies based on federated learning. Traditional federated learning typically performs weighted aggregation of the global model based on the performance of each center’s model (32). This approach may lead to the global model overfitting the data distribution of certain centers with easily distinguishable data categories, while neglecting the more complex and diverse data from other centers, thus limiting the global model’s performance. Another common method is to allocate weights based on the data volume of participating centers (18). Although this method emphasizes the importance of data scale, it overlooks differences in data quality. Redundant information at the local level can negatively impact the global model and make data contribution evaluation less accurate.

Research on fair federated learning models has made some progress in recent years but still has many shortcomings. Hosseini et al. (33) proposed Prop-FFL, which incorporates fairness constraints into the optimization objective to reduce performance gaps between different participants. Although this method partially alleviates fairness issues caused by non-independent and identically distributed (non-IID) data, its optimization objective overly emphasizes balance, potentially sacrificing the global model’s performance. Jiang et al. (34) introduced the FedCE algorithm, which estimates client contributions in both gradient and data spaces, using these estimates to assign aggregation weights while considering both model performance fairness and collaboration fairness. However, in its evaluation of contributions in the data space, this method still fails to account for redundant information that offers low or even negative contributions to the global model. Relying solely on data volume as a metric makes it difficult to evaluate each client’s data contribution accurately and fairly, while redundant information can limit the overall model performance.

To address the limitations of traditional federated learning and existing fair federated frameworks, this study proposes a Contribution-Fair Federated Learning model based on multi-center core data extraction (FedCMC). The test set AUCs for Centers A, B, and C reached 0.8261, 0.8750, and 0.8964, respectively, demonstrating good generalization performance across centers. Compared with traditional algorithms such as FedAvg, Moon, and FedProx, FedCMC achieved the highest AUC performance at all three centers. On average, the performance and model fairness improved by 10.61% and 31.24%, respectively, compared to traditional federated learning algorithms (details in **Supplementary Table S7**). These findings indicate that FedCMC not only prioritizes fairness in multi-center medical settings, but also significantly enhances the diagnostic performance for predicting myometrial invasion status in endometrial cancer patients.

Compared with other federated algorithms, FedCMC has the following advantages: 1) More accurate and fair contribution evaluation. This study comprehensively evaluates each center’s contribution based on data information richness and model quality. At the data level, instead of using data size to evaluate contributions, an innovative MCDEM was designed. The MCDEM eliminates redundant data, selecting core datasets for each center. By reducing the over-representation of redundant information, the model focuses on under-represented data, improving generalization while enabling fairer contribution evaluations. After processing with the MCDEM, the core datasets of Centers A, B, and C accounted for 57.4%, 43.5%, and 88.7% of the original data, respectively, removing 43.4% of redundant data overall. The first and second groups in the ablation experiment in **Table 4** show that using only the core datasets for model training improved the AUCs of the three centers by 3.66%, 2.61%, and 2.25%, respectively, fully validating the effectiveness of MCDEM. At the model level, the local model accuracy was used to evaluate its contribution, emphasizing high-quality models and reducing the adverse impact of low-quality models on the global model. 2) Fair and reasonable reward distribution through weighted aggregation based on two fairness metrics, D and Q. High-contribution centers often possess richer data information and better model quality. Increasing their weight in the global model aggregation improves overall performance and aligns the global model more closely with the data distribution of high-contribution centers. This ensures that high-contribution centers gain more significant performance benefits, establishing a fair and reasonable federated incentive mechanism to address collaboration fairness issues. Analysis of the second and third groups in the ablation experiment in **Table 4** shows that weighted aggregation based on the two fairness metrics significantly improved the performance of high-contribution centers, with Center A showing the most pronounced improvement—a 5.40% increase in AUC. 3) A simple and effective personalized federated learning strategy. Each local endpoint fine-tunes the model after acquiring prior knowledge from the global model. This improves the model’s adaptability to local data, mitigating inconsistencies in model performance under highly heterogeneous data scenarios.

Existing reviews categorize fairness challenges in federated learning into collaboration fairness and model fairness (35, 36). From the perspective of collaboration fairness, FedCMC improves the benefits for high-contribution centers through accurate contribution evaluation at the data and model levels and fair aggregation based on contributions, enhancing multi-center collaboration incentives. From the perspective of model fairness, FedCMC improves performance consistency compared to three traditional federated learning algorithms through a simple personalized federated learning strategy. Additionally, we believe that collaboration fairness and model fairness are not entirely independent but mutually reinforcing under a fair federated framework. Addressing collaboration fairness promotes consistency in model performance, while tackling model fairness in turn incentivizes more institutions to participate in federated learning. This mutual reinforcement is crucial for building more robust federated learning models, thereby offering stronger support for clinical preoperative prediction and personalized treatment.

Despite the achievements of this study, there are still limitations. The current research mainly focuses on client-level fairness and has not deeply explored attribute-level fairness at the sample level (35), nor has it investigated the issue of communication overhead in federated learning. Additionally, the dataset used in this study is relatively limited in scale. Future research could explore attribute-level fairness based on larger and more diverse datasets to improve the existing fairness framework and design more robust and fair federated learning models.

## 5 Conclusion

To address preoperative myometrial invasion prediction in endometrial cancer and fairness concerns in medical federated learning, we propose FedCMC, a contribution-fair federated learning model based on multi-center core data extraction. By more accurately and fairly quantifying each center’s contribution at both the data and model levels, and employing a contribution-based adaptive aggregation strategy, FedCMC places greater emphasis on high-contributing centers to enhance fairness and incentivize broader participation. Experiments on preoperative myometrial invasion prediction demonstrate that FedCMC yields more pronounced performance gains for high-contributing centers. Compared with three traditional federated learning algorithms, FedCMC not only alleviates fairness issues in federated learning but also enhances classification performance in preoperative myometrial invasion prediction, offering potential technical support for personalized treatment of EC patients.

## Data Availability

The original contributions presented in the study are included in the article/Supplementary Material. Further inquiries can be directed to the corresponding authors (jmlws2@163.com).
