# Supplementary material for: FedCMC: a federated learning model with contribution fairness based on multi-center core data extraction for assessing the myometrial invasion status of endometrial cancer
Source: Front Oncol. 2025 Sep 9;15:1648502. doi: 10.3389/fonc.2025.1648502 (PMC12454072; doi:10.3389/fonc.2025.1648502)
Supplement: Supplementary file 1 [file DataSheet1.docx]

**Supplemental Materials**

**Supplementary S1: Data preprocessing**

The preprocessing steps for MRI images are shown in Figure S1. First, doctors identify the rectangular regions of interest (ROIs) from the original MRI images from four data centers. The ROIs are carefully selected to encompass the entire contour of the lesion. Subsequently, the size of the ROI images is adjusted to a rectangular shape of 224×224. Finally, each data center contributes a different number of ROIs: centers A, B, and C contribute 6422, 3320, and 1141 ROIs, respectively.

**Supplementary S2: Multi-center core data extraction module**

Before conducting federated learning, we designed a multi-center core data extraction module to remove samples with high information redundancy. Specifically, we used a trained model to predict the samples in the validation set and measured the prediction error using mean absolute error (MAE). If the error is lower than the threshold $\tau_{e}$, it indicates that the information of the sample is already well represented in the training data. Therefore, such samples are considered redundant and excluded from model training.

The iterative process of data selection is illustrated in Figure 1b in the main text. Starting with the complete training set, we iteratively reduce the size of the training set. The initial local training pool at each medical center is denoted as $S_{0}$, the training set in the ith iteration as $T_{i}$, the validation set as $V_{i}$, the identified redundant dataset as $R_{i}$, and the trained model as $M_{i}$. Taking the first iteration (i = 1) as an example: first, $S_{0}$ is randomly split into a training set $T_{1}$ (80% of $S_{0}$) and a validation set $V_{1}$ (20% of $S_{0}$). Then, the model $M_{1}$ is trained on $T_{1}$ and validated on $V_{1}$. Next, samples in $V_{1}$ with prediction errors below the threshold are removed from $S_{0}$, forming $R_{1}$, and the updated training pool becomes $S_{1}=S_{0}-R_{1}$. In subsequent iterations, samples in $R_{1}$ are no longer used, and the parameters of model $M_{1}$ are not inherited. After n iterations, we obtain a redundant data set containing highly redundant information and an core set representing the diverse information richness of each medical center.

**Supplementary S3: Contribution fairness aggregation mechanism**

The contribution fairness aggregation process is illustrated in Figure S2. The set of all centers participating in federated learning is denoted as $\psi$, and the update formula for the global model is shown in Equation (1):

$G=\sum_{k\in\psi} w_{k}M_{k}$ (1)

In the equation, $G$ represents the global model parameters, $M_{k}$ represents the local model parameters of center k in the current round, and $w_{k}$ represents the aggregation weight of center k. The weight $w_{k}$ is determined based on the model quality $Q_{k}$ and data richness $D_{k}$ of center k, where $Q_{k}$ denotes the accuracy of the local model at center k, and $D_{k}$ denotes the number of effective samples at center k after being processed by the multi-center core data extraction module (MCDEM). The calculation process for $w_{k}$ is given in Equation (2):

$w_{k}=\frac{\alpha w_{d_{k}}+(1-\alpha)w_{q_{k}}}{\sum_{k\in\psi} (\alpha w_{d_{k}}+(1-\alpha)w_{q_{k}})}$ (2)

In the equation, $\alpha$ is a balancing parameter for contribution evaluation, used to adjust the emphasis on data richness and model quality when assessing the contribution of each center.

Equations (3) and (4) respectively calculate the weight coefficients for model quality and data richness of center k.

$w_{q_{k}}=\frac{Q_{k}}{\sum_{k\in\psi} Q_{k}}$ (3)

$w_{d_{k}}=\frac{D_{k}}{\sum_{k\in\psi} D_{k}}$ (4)

**Supplementary S4: Sparse Bayesian extreme learning machine**

Sparse Bayesian extreme learning machine (SBELM) is an algorithm for solving single-hidden-layer feedforward networks. Its main characteristics include randomly assigning the connection weights $\omega$ and biases $b$ between the input and hidden layers, and generating the corresponding output matrix simply by setting the network structure. This approach offers faster learning speed and better generalization ability. The training objective function is shown in Equation (5):

$\mathrm{argmin}E(\omega,b)=\left\|y-X\omega\right\|^{2}+\lambda\sum_{i-1}^{L} \left\|\omega_{i}\right\|_{1}$ (5)

In the equation, $y$ represents the true labels of the samples, $X$ represents the output of the hidden layer, $\omega$ denotes the weight vector between the hidden layer and the output layer, $L$ is the number of neurons in the hidden layer, and $\lambda>0$ is the $L_{1}$-norm regularization coefficient. To handle noise in the output and achieve feature selection, the likelihood function of $\omega$ is defined as follows:

$P(y\left|X,\omega,\beta\right.)=N(y\left|X^{T}\omega,\beta^{-1}\right.)$ (6)

In this equation, the noise is assumed to follow a zero-mean Gaussian random variable with an inverse variance $\beta$, and $y$ is expressed as a linear combination of Gaussian noise. To obtain the posterior probability of $\omega$, a sparse prior for $\omega$ is introduced, as shown in Equation (7):

$P(\omega\left|\alpha\right.)=\prod_{k=1}^{L} N(\omega_{i}\left|0,\alpha_{i}^{-1}\right.)$ (7)

Here, $\alpha=[\alpha_{1},\alpha_{1},...,\alpha_{L}]^{T}$ represents the independent prior parameter for each weight vector $\omega_{i}$. Based on Bayes' theorem, the posterior probability of the weight vector $\omega$ is given as:

$P(\omega\left|y,X,\alpha,\beta\right.)=N(\omega\left|m,\sum\right.)$ (8)

The posterior mean $m$ and covariance matrix are expressed as shown in Equation (9):

$\left\{\begin{matrix} m=\beta\sum X^{T}y \\ \sum^{-1}=diag(\alpha)+\beta X^{T}X \end{matrix}\right.$ (9)

The hyperparameters $\alpha_{i}$ and $\beta$ are updated using the marginal likelihood estimation method, with the specific iterative formula provided in Equation (10):

$\left\{\begin{matrix} a_{i}^{j+1}=\frac{1-a_{i}^{j}\sum_{ii}}{m_{i}^{T}m_{i}} \\ (\beta^{j+1})^{-1}=\frac{\left\|y-Xm\right\|^{2}}{N-\sum_{i=1}^{L} (1-a_{k}^{j}\sum_{kk})} \end{matrix}\right.$ (10)

In this equation, $m_{i}$ is the ith component of the posterior mean $m$, and is the ith diagonal component of the posterior covariance .

To maximize the log-likelihood, the optimization is solved as follows: Given the initial values of $\alpha$ and $\sigma^{2}$, the Gaussian distribution mean $m$ and covariance are iteratively solved using Equation (9). The convergence of $\omega$ is then checked. If it does not converge, the parameters $\alpha$ and $\beta$ are updated using Equation (10), and the mean $m$ and covariance are recalculated. If convergence is achieved, then $\omega=m$. During this process, some elements of $\alpha$ approach infinity, causing the corresponding components of $\omega$ to have a zero posterior distribution. Finally, the nonzero vector of $\omega$ is used to construct the model and perform predictions.

**Figure S1**


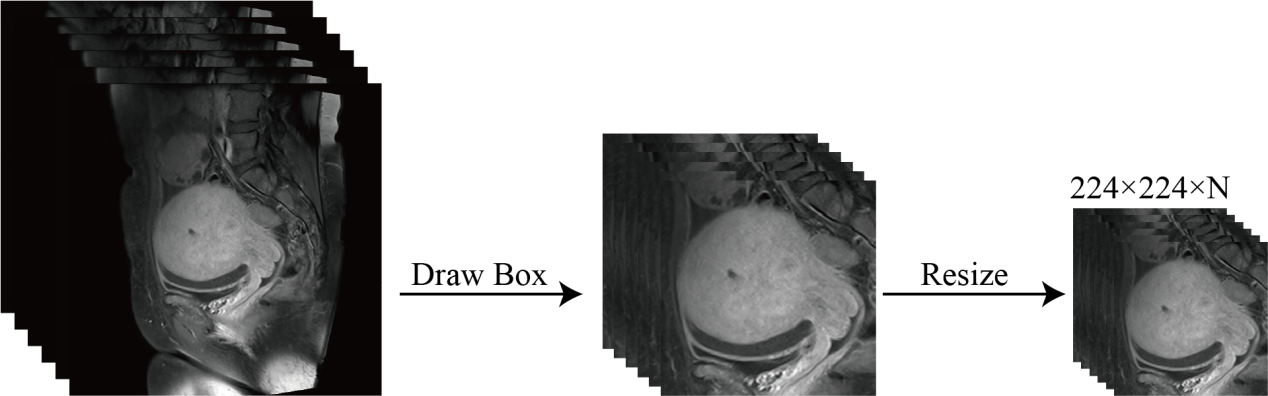


The preprocessing procedure for MRI images of a single patient is illustrated. Note: N represents the number of MRI images belonging to the patient.

**Figure S2**


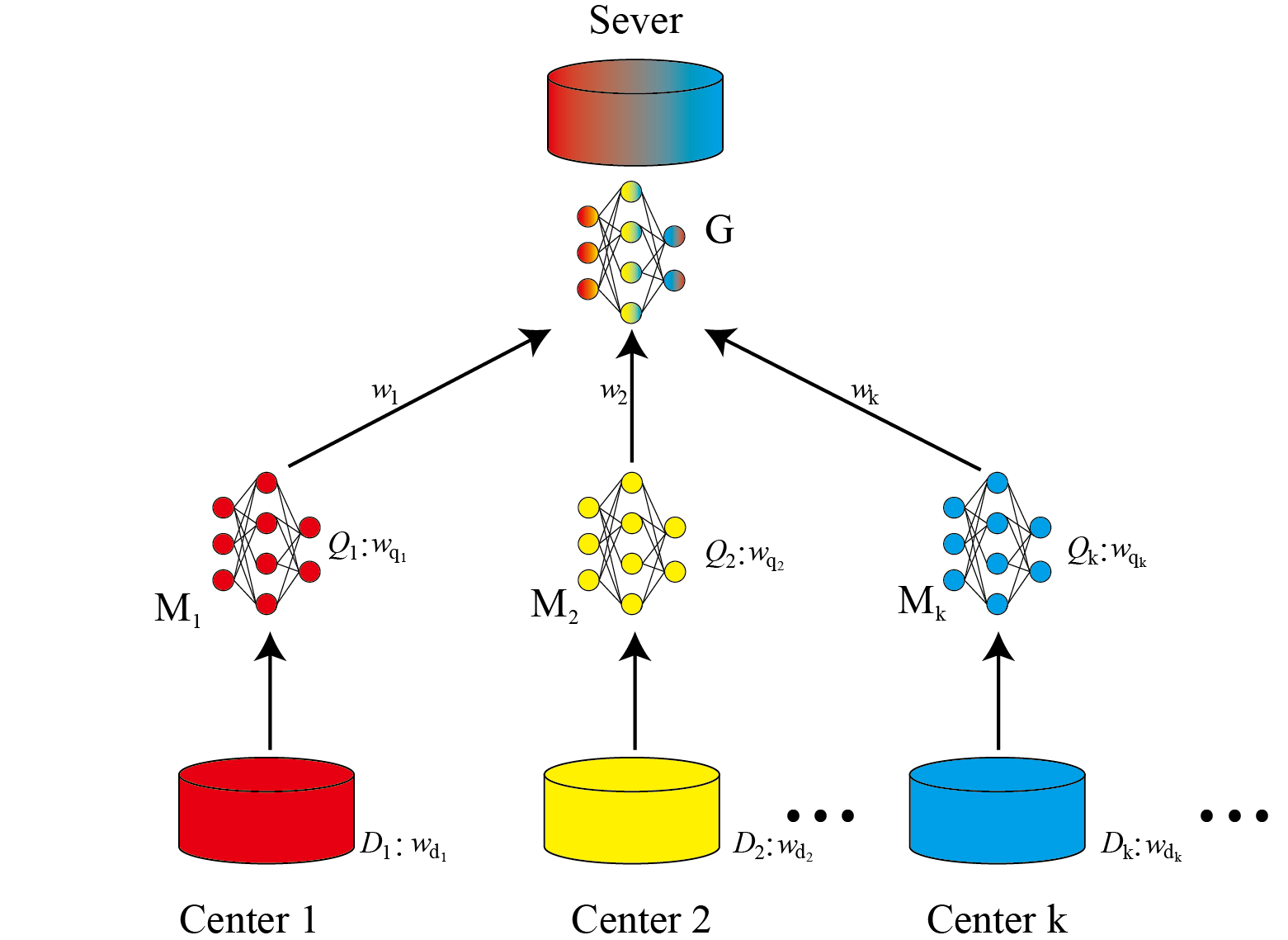


The above figure demonstrates the process of aggregating local models from different centers into a global model. Note: $D_{k}$ represents the data richness of each center, and $w_{d_{k}}$ denotes its corresponding weight coefficient. $Q_{k}$ indicates the model quality of the local model $M_{k}$, and $w_{q_{k}}$ represents its corresponding weight coefficient. $w_{k}$ denotes the aggregation weight of the local model from center k. $G$ represents the global model.

**Figure S3**


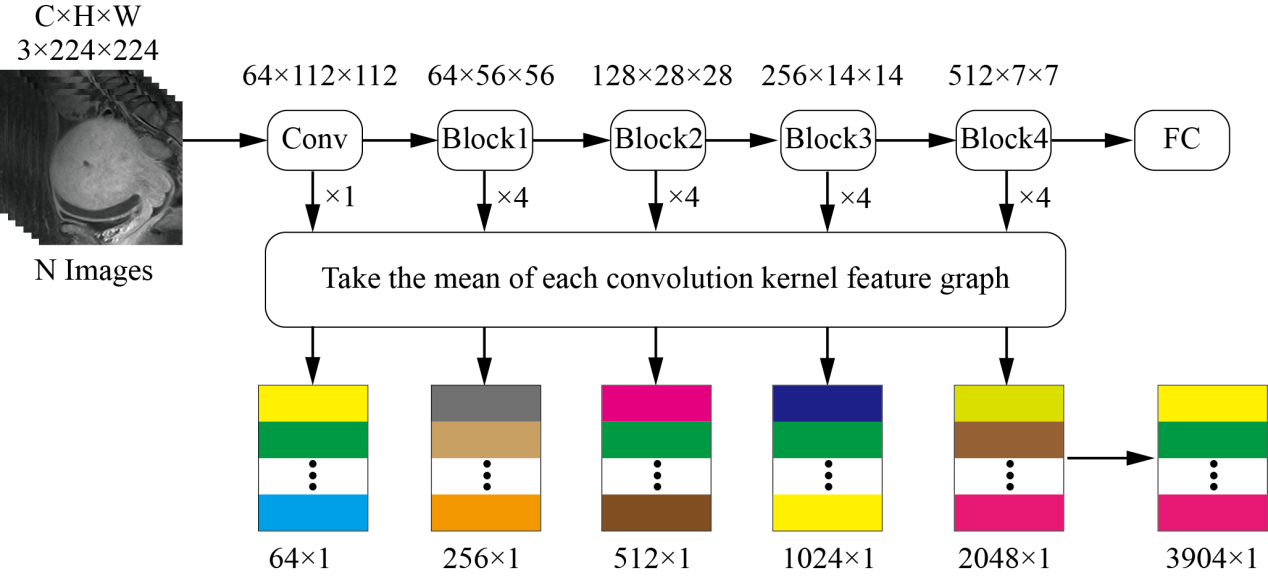


This figure depicts the process of feature extraction from patients' MRI images. In this study, the ResNet18 model is employed for feature extraction. Since the feature extraction network contains 3904 convolutional filters, 3904 features are extracted from each image. Features from the same filter are averaged across N instances. As a result, each patient is associated with a matrix containing 3904 features. Note: Conv: Convolutional layer. Block1 to Block4 represent the main network of ResNet18. FC: Fully connected layer.

**Figure S4**


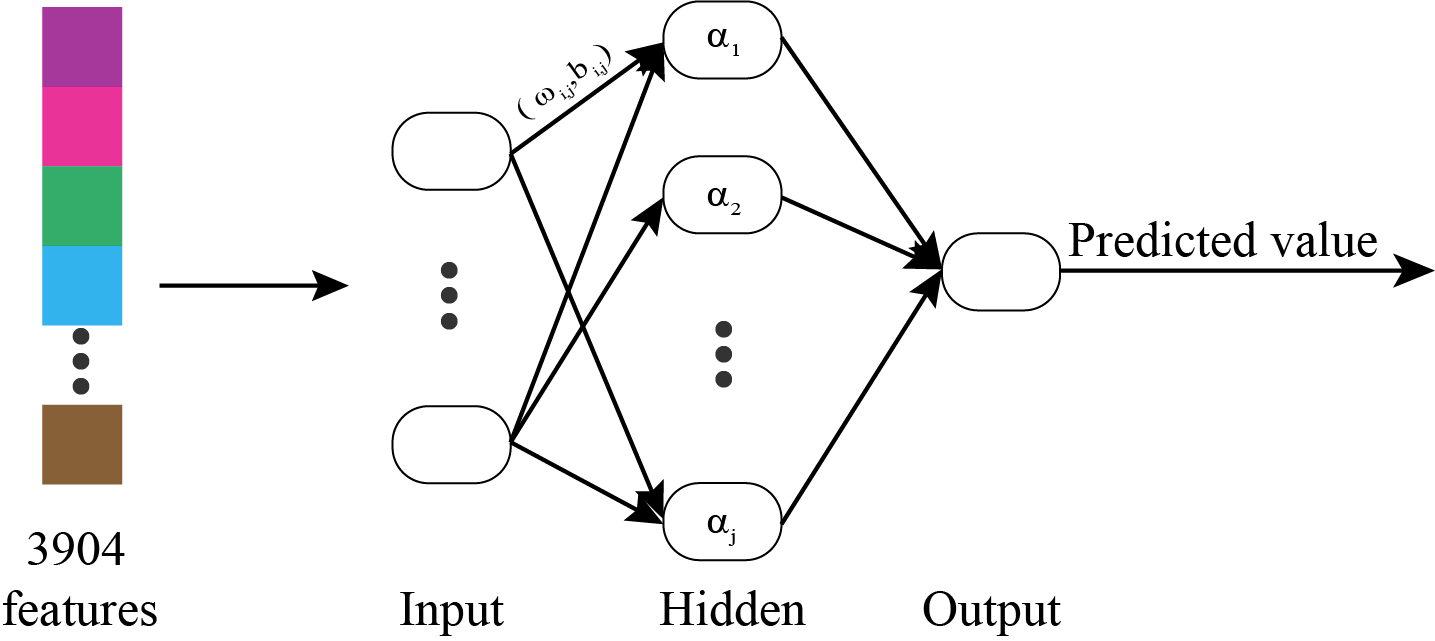


The above figure illustrates the classifier's processing of extracted features. A total of 3904 features for each patient are input into the Sparse Bayesian Extreme Learning Machine (SBELM) to obtain the final model predictions. Note: $\omega_{i,j}$ represents the connection weights between the input layer and the hidden layer. $b$ denotes the biases between the input layer and the hidden layer. $\alpha_{j}$ refers to the parameters of the hidden layer neurons.

**Table S1. MR imaging sequences and parameters of Center A**

| Vendor | Field Strength (T) | Scan Type | Sequence | Acquisition Plane | TR/TE (msec) | Reconstruction Matrix | NEX | FOV (cm) | Slice Thickness(mm) | Slice Gap(mm) | b-values (s/mm) |
| --- | --- | --- | --- | --- | --- | --- | --- | --- | --- | --- | --- |
| Ingenia (Philips, Holland) | 3 | T2W | TSE | Sagittal | 2317/102 | 352×352 | 1 | 28×28 | 4 | 0.4 | 0 and 1000 |
|  | 3 | T2W | TSE | Axial | 4383/122 | 300×300 | 1 | 36×36 | 4 | 1 |  |
|  | 3 | T2W | TSE | Coronal | 2359/97 | 380×380 | 1 | 38×38 | 4 | 1 |  |
|  | 3 | T1W | 3D FFE | Axial | 4.3/1.5 | 328×258 | 1 | 36×28 | 4 | 0 |  |
|  | 3 | DW | SE-EPI | Axial | 2367/75 | 128×124 | 2 | 38×38 | 4 | 0 |  |
|  | 3 | T1W+C | 3D FFE | Axial | 4.3/1.5 | 328×258 | 1 | 36×28 | 4 | 0 |  |
|  | 3 | T1W+C | 3D FFE | Sagittal | 4.3/1.5 | 228×227 | 1 | 25×25 | 2 | -2 |  |
|  | 3 | T1W+C | 3D FFE | Coronal | 4.3/1.5 | 236×330 | 1 | 26×36 | 2 | -2 |  |
| Espree (Siemens, Germany) | 1.5 | T2W | TSE | Sagittal | 3150/100 | 320×320 | 1 | 28×28 | 4 | 1.2 | 0 and 800 |
|  | 1.5 | T2W | TSE | Axial | 4330/56 | 320×320 | 3 | 37×37 | 6 | 1.2 |  |
|  | 1.5 | T2W | TSE | Coronal | 3150/100 | 320×320 | 1 | 35×35 | 4 | 1.2 |  |
|  | 1.5 | T1W | TSE | Axial | 900/7.4 | 320×320 | 4 | 37×37 | 6 | 1.2 |  |
|  | 1.5 | DW | SE-EPI | Axial | 6500/86 | 170×170 | 6 | 28×28 | 6 | 1.2 |  |
|  | 1.5 | T1W+C | 3D FLASH | Axial | 7.07/2.39 | 320×320 | 1 | 38×38 | 5 | 1 |  |
|  | 1.5 | T1W+C | TSE | Sagittal | 620/7.7 | 320×320 | 6 | 28×28 | 4 | 1.2 |  |
|  | 1.5 | T1W+C | TSE | Coronal | 725/8.1 | 320×320 | 4 | 30×30 | 4 | 1.2 |  |

Note: T2W = T2-weighted; T1W = T1-weighted; DW = diffusion-weighted; T1W+C = T1-weighted contrast-enhanced; TR = repetition time; TE = echo time; NEX = number of excitations; FOV = field of view; FSE = fast spin echo; SE = spin echo; EPI = echo planar imaging; FFE = fast field echo; FLASH = fast low angle shot.

**Table S2. MR imaging sequences and parameters of Center B**

| Vendor | Field Strength (T) | Scan Type | Sequence | Acquisition Plane | TR/TE (msec) | Base resolution | NEX | FOV (cm) | Slice Thickness(mm) | Slice Gap(mm) | b-values (s/mm) |
| --- | --- | --- | --- | --- | --- | --- | --- | --- | --- | --- | --- |
| Siemens (MAGNETOM Vida) | 3 | DW | ep2d_diff_b0_1500 | Transverse | 6200/69 | 164 | 2 | 38×38 | 4 | 0.4 | 0，1000 and 1500 |
|  | 3 | T2W | TSE_DIXON | Transverse | 4700/95 | 384 | 2 | 27×27 | 4 | 0.4 |  |
|  | 3 | T2W | TSE | Sagittal | 4500/101 | 320 | 2 | 24×24 | 4 | 0.4 |  |
|  | 3 | T2W | TSE_FS | Coronal | 4500/101 | 320 | 2 | 26×26 | 4 | 0.4 |  |
|  | 3 | T1W | QTSE | Transverse | 600/9.5 | 448 | 1 | 36×36 | 4 | 0.4 |  |
|  | 3 | T1W pre-contrast | YIBE_DIXON | Transverse | 5.5/2.46 | 352 | 1 | 38×38 | 3 | 0.6 |  |
|  | 3 | T1W+C | YIBE_DIXON | Transverse | 5.5/2.46 | 352 | 1 | 37×37 | 3 | 0.6 |  |
|  | 3 | T1W+C | YIBE_DIXON | Sagittal | 5.6/2.46 | 352 | 1 | 38×38 | 3 | 0.6 |  |
|  | 3 | T1W+C | YIBE_DIXON | Coronal | 5.6/2.46 | 352 | 1 | 30×30 | 3 | 0.6 |  |

Note: T2W = T2-weighted; T1W = T1-weighted; DW = diffusion-weighted; T1W+C = T1-weighted contrast-enhanced; TR = repetition time; TE = echo time; NEX = number of excitations; FOV = field of view; FSE = fast spin echo; SE = spin echo; EPI = echo planar imaging; FFE = fast field echo; FLASH = fast low angle shot.

**Table S3. MR imaging sequences and parameters of Center C**

| Vendor | Field Strength (T) | Scan Type | Sequence | Acquisition Plane | TR/TE (msec) | Reconstruction Matrix | NEX | FOV (cm) | Slice Thickness(mm) | Slice Gap(mm) | b-values (s/mm) |
| --- | --- | --- | --- | --- | --- | --- | --- | --- | --- | --- | --- |
| Ingenia (Siemens, skyra) | 3 | T2W | TSE | Sagittal | 2800/100 | 320×280 | 1 | 30×21 | 4 | 0.4 | 0 and 1000 |
|  | 3 | T2W | TSE | Axial | 4300/122 | 360×320 | 1 | 38×38 | 4 | 1 |  |
|  | 3 | T2W | TSE | Coronal | 2359/97 | 380×380 | 1 | 38×38 | 4 | 1 |  |
|  | 3 | T1W | 3D FFE | Axial | 4.3/1.3 | 320×268 | 1 | 38×38 | 4 | 0 |  |
|  | 3 | DW | SE-EPI | Axial | 2500/80 | 128×124 | 2 | 38×38 | 4 | 0 |  |
|  | 3 | T1W+C | 3D FFE | Axial | 4.5/1.3 | 258×228 | 1 | 38×38 | 3 | 0 |  |
|  | 3 | T1W+C | 3D FFE | Sagittal | 4.5/1.3 | 268×258 | 1 | 38×38 | 3 | 0 |  |
|  | 3 | T1W+C | 3D FFE | Coronal | 4.5/1.3 | 258×228 | 1 | 38×38 | 3 | 0 |  |
| Espree (Siemens, Germany) | 1.5 | T2W | TSE | Sagittal | 3150/100 | 320×320 | 1 | 28×28 | 4 | 0 | 0 and 800 |
|  | 1.5 | T2W | TSE | Axial | 4000/58 | 320×320 | 3 | 37×37 | 6 | 1.2 |  |
|  | 1.5 | T2W | TSE | Coronal | 3200/100 | 320×320 | 1 | 36×36 | 4 | 1.2 |  |
|  | 1.5 | T1W | TSE | Axial | 750/7.1 | 320×320 | 4 | 37×37 | 6 | 1.2 |  |
|  | 1.5 | DW | SE-EPI | Axial | 5800/75 | 170×170 | 6 | 28×28 | 5 | 1.2 |  |
|  | 1.5 | T1W+C | TSE | Axial | 680/7.9 | 320×320 | 1 | 32×32 | 4 | 1.2 |  |
|  | 1.5 | T1W+C | TSE | Sagittal | 620/7.7 | 320×320 | 4 | 28×28 | 4 | 1.2 |  |
|  | 1.5 | T1W+C | TSE | Coronal | 725/8.1 | 320×320 | 4 | 30×30 | 4 | 1.2 |  |

Note: T2W = T2-weighted; T1W = T1-weighted; DW = diffusion-weighted; T1W+C = T1-weighted contrast-enhanced; TR = repetition time; TE = echo time; NEX = number of excitations; FOV = field of view; FSE = fast spin echo; SE = spin echo; EPI = echo planar imaging; FFE = fast field echo; FLASH = fast low angle shot.

**Table S4. MR imaging sequences and parameters of Center D**

| Vendor | Field Strength (T) | Scan Type | Sequence | Acquisition Plane | TR/TE (msec) | Reconstruction Matrix | NEX | FOV (cm) | Slice Thickness(mm) | Slice Gap(mm) | b-values (s/mm) |
| --- | --- | --- | --- | --- | --- | --- | --- | --- | --- | --- | --- |
| GE-SIGNA Architect | 3 | T2W | TSE | Sagittal | 3127/123 | 288×288 | 1 | 22×22 | 4 | 5 | 0 and 1000 |
|  | 3 | T2W | TSE | Axial | 3038/80 | 320×320 | 2 | 32×32 | 5 | 7 |  |
|  | 3 | T2W | TSE | Coronal | 3699/77 | 300×200 | 1 | 26×39 | 5 | 6 |  |
|  | 3 | T1W | 3D FFE | Axial | 688/7 | 360×280 | 1 | 32×32 | 5 | 7 |  |
|  | 3 | DW | SE-EPI | Axial | 6835/76 | 140×168 | 1 | 32×32 | 5 | 7 |  |
|  | 3 | T1W+C | 3D FFE | Axial | 3.5/1.5 | 288×212 | 1 | 40×40 | 5 | 5 |  |
|  | 3 | T1W+C | 3D FFE | Sagittal | 4.0/1.7 | 288×212 | 1 | 28×28 | 5 | 5 |  |
|  | 3 | T1W+C | 3D FFE | Coronal | 3.5/1.6 | 288×212 | 1 | 32×32 | 5 | 5 |  |
| Philips  Achieva | 1.5 | T2W | TSE | Sagittal | 5120/90 | 244×198 | 4 | 23×23 | 5 | 5.5 | 0 and 800 |
|  | 1.5 | T2W | TSE | Axial | 3327/80 | 236×208 | 4 | 23×35 | 7.5 | 8.5 |  |
|  | 1.5 | T2W | TSE | Coronal | 3327/80 | 212×143 | 3 | 23×35 | 5 | 5.5 |  |
|  | 1.5 | T1W | TSE | Axial | 495/10 | 260×243 | 2 | 30×35 | 7.5 | 8.5 |  |
|  | 1.5 | DW | SE-EPI | Axial | 3000/64 | 136×104 | 3 | 34×26 | 7.5 | 8.5 |  |
|  | 1.5 | T1W+C | 3D FLASH | Axial | 467/10 | 260×227 | 2 | 40×25 | 7.5 | 8.5 |  |
|  | 1.5 | T1W+C | TSE | Sagittal | 469/10 | 220×192 | 2 | 23×23 | 5 | 6 |  |
|  | 1.5 | T1W+C | TSE | Coronal | 468/8 | 220×162 | 1 | 23×35 | 5 | 5.5 |  |

Note: T2W = T2-weighted; T1W = T1-weighted; DW = diffusion-weighted; T1W+C = T1-weighted contrast-enhanced; TR = repetition time; TE = echo time; NEX = number of excitations; FOV = field of view; FSE = fast spin echo; SE = spin echo; EPI = echo planar imaging; FFE = fast field echo; FLASH = fast low angle shot.

**Table S5. Test set results of the four centers using different classifiers based on the FedCMC framework**

| Classifier | Center A | Center B | Center C |
| --- | --- | --- | --- |
| SBELM | **0.8261/0.7800** | **0.8750/0.8729** | **0.8964/0.9535** |
| ELM | 0.7513/0.7350 | 0.8273/0.7542 | 0.8649/0.7209 |
| K_NN | 0.5666/0.5150 | 0.7653/0.5678 | 0.8041/0.9302 |
| MLP | 0.6281/0.6400 | 0.6602/0.7797 | 0.7613/0.6977 |
| SVM | 0.5719/0.6800 | 0.7216/0.7458 | 0.7883/0.7674 |

Note: The values on either side of the ‘/’ represent AUC and ACC respectively (i.e., AUC/ACC). Bold font indicates the best metric in each column. Classifiers evaluated include SBELM (Sparse Bayesian Extreme Learning Machine), ELM (Extreme Learning Machine), K_NN (K-Nearest Neighbors), MLP (Multi-layer Perceptron), and SVM (Support Vector Machine).

**Table S6. DeLong, NRI, and IDI comparison between FedCMC and other federated learning algorithms on the test set**

|  | | | Fedavg | Moon | Fedprox |
| --- | --- | --- | --- | --- | --- |
| FedCMC | Center A | DeLong | 1.7818  (p = 0.0748) | 1.9387  (p = 0.0525) | 1.2365  (p = 0.2163) |
|  |  | NRI | **0.6629**  **(p = 0.0009)** | **0.6286**  **(p = 0.0012)** | **0.5257**  **(p = 0.0105)** |
|  | Center B | DeLong | 1.6686  (p = 0.0952) | 0.9658  (p = 0.3341) | 0.8165  (p = 0.4142) |
|  |  | NRI | **0.8091**  **(p = 0.0113)** | **1.0409**  **(p < 0.0001)** | **0.7000**  **(p = 0.0288)** |
|  | Center C | DeLong | 0.7831  (p = 0.4336) | 0.2935  (p = 0.7692) | 0.4408  (p = 0.6593) |
|  |  | NRI | **1.2342**  **(p = 0.0002)** | **0.9099**  **(p = 0.0081)** | **0.9099**  **(p = 0.0081)** |

Note: Bolded values indicate statistically significant differences with p < 0.05.

**Table S7. Improvements of FedCMC over traditional federated learning algorithms in terms of performance and fairness**

| Algorithm | mean±std | Improvement | |
| --- | --- | --- | --- |
|  |  | performance | fairness |
| Fedavg | 0.7508±0.0429 | 15.31% | 31.24% |
| Moon | 0.8008±0.0455 | 8.12% | 35.17% |
| Fedprox | 0.7981±0.0406 | 8.41% | 27.32% |
| FedCMC | 0.8658±0.0295 | 0% | 0% |

Note: Mean and standard deviation are used to represent model performance and inconsistency (i.e., fairness), respectively. Relative growth rate is applied to quantify the improvement of FedCMC compared to the other three federated learning algorithms.
